# Estimation of nephron number in living humans by combining unenhanced computed tomography with biopsy-based stereology

**DOI:** 10.1038/s41598-019-50529-x

**Published:** 2019-10-07

**Authors:** Takaya Sasaki, Nobuo Tsuboi, Yusuke Okabayashi, Kotaro Haruhara, Go Kanzaki, Kentaro Koike, Akimitsu Kobayashi, Izumi Yamamoto, Sho Takahashi, Toshiharu Ninomiya, Akira Shimizu, Andrew D. Rule, John F. Bertram, Takashi Yokoo

**Affiliations:** 1 Division of Nephrology and Hypertension, Department of Internal Medicine, The Jikei University School of Medicine, Tokyo, Japan; 2 Clinical Research Support Center, The Jikei University School of Medicine Tokyo, Tokyo, Japan; 3 Department of Epidemiology and Public Health, Graduate School of Medical Sciences, Kyushu University, Fukuoka, Japan; 4 Department of Analytic Human Pathology, Nippon Medical School, Tokyo, Japan; 5 Division of Nephrology and Hypertension, Mayo Clinic, Rochester, Minnesota USA; 6 Department of Anatomy and Developmental Biology and Biomedical Discovery Institute, Monash University, Melbourne, Australia

**Keywords:** Glomerulus, Kidney diseases

## Abstract

Methods for estimating nephron number in a clinical setting may be useful for predicting renal outcomes. This study aimed to establish such a method using unenhanced computed tomography (CT) and biopsy-based stereology. Patients or living kidney donors simultaneously subjected to enhanced and unenhanced CT examinations were randomly assigned to development and validation groups. The enhanced CT-measured arterial phase and the venous phase images of kidneys were regarded as the true values for cortical volume and parenchymal volume, respectively. Linear multiple regression analysis was used to create models for estimating cortical volume using explanatory variables including unenhanced CT-measured parenchymal volume. Nephron number was determined as the product of cortical volume and the glomerular density in biopsies of donors. Five equations for estimating cortical volume were created and verified. In donors, estimated nephron number by unenhanced CT was consistent with that by enhanced CT, with minimal errors in all models (636–655 ± 210–219 vs. 648 ± 224 × 10^3^/kidney). Clinical characteristics combined with parenchymal volume did not improve the equation over parenchymal volume alone. These results support the feasibility of estimating nephron number by a combination of unenhanced CT and biopsy-based stereology, with a possible application for renal disease patients who are often not suitable for contrast media.

## Introduction

Renal cortical volume has previously been used as a surrogate measure of renal function^1–3^. Recent studies have reported a method for measuring cortical volume using images of contrast-enhanced computed tomography (CT)^4–8^. With this method, the intensified part of the arterial phase following injection of contrast medium is regarded as cortical volume. By combining enhanced CT-measured cortical volume and glomerular numerical density obtained using stereological analysis of implantation biopsies from American living kidney donors, a method for estimating total nephron number per kidney was proposed^9^. The findings were consistent with those in previously reported autopsy series of American Caucasians obtained using the disector/fractionator method, the current gold-standard method for estimating total nephron number^9,10^. We recently used a combined CT angiography and biopsy-based approach to estimate nephron number in Japanese living kidney donors^11^. The estimated total nephron number obtained in the study was quite similar to that reported in a previous Japanese autopsy study using the disector/fractionator method, again confirming feasibility of the method^11,12^.

Despite the potential utility of estimating cortical volume using CT angiography in the clinical setting, the method has a serious disadvantage in that the contrast media is not preferable for the majority of patients with renal disease and/or renal impairment. In the present study, we therefore aimed to create a regression equation for estimating cortical volume using unenhanced CT-measured parenchymal volume, which can be applied to renal disease patients. In addition, we validated the method for estimating nephron number by combining an equation-based cortical volume with biopsy-based stereology.

## Results

### Clinical and histopathological characteristics

This study included 49 kidney donors and 58 subjects with renal disease. Clinical characteristics of the subjects are provided in Table 1. Overall, mean subject age was 59.1 years, about half of the subjects were male, and approximately half of the subjects had a history of hypertension. The indications for enhanced CT examinations in diseased patients are listed in Supplemental Table S1. The 107 subjects were randomly assigned at a ratio of 3:1 to either a derivation group for model creation (N = 80) or a validation group for verification (N = 27). There were no significant differences in the clinical characteristics or measurement results between the derivation and validation groups (Supplemental Table S2).

**Table 1:** Clinical characteristics and volumetric measurements (N = 107).

	All subjects
	N = 107
Donor, N (%)	49 (45.8)
Age, years	59.1 ± 12.6
Male, N (%)	50 (46.7)
Hypertension, N (%)	54 (50.5)
Duration of hypertension, years	9 (4–12)
Diabetes mellitus, N (%)	17 (15.9)
Duration of diabetes mellitus, years	3 (2–8)
Obesity, N (%)	37 (34.6)
CKD stage G1 and G2, N (%)	65 (60.7)
CKD stage G3, N (%)	36 (33.6)
CKD stage G4 and G5, N (%)	6 (5.6)
Body weight, kg	61.3 ± 12.1
Body height, cm	161.7 ± 7.9
Cr, mg/dL	0.80 (0.68–1.02)
eGFR, ml/min/1.73 m^2^	64.4 ± 19.9
Albumin, mg/dL	3.98 ± 0.58
Serum total cholesterol, mg/dL	203 ± 39
Hemoglobin A1c, %	5.6 ± 0.8

Abbreviations: CKD, chronic kidney disease; Cr, creatinine; eGFR, estimated glomerular filtration rate.

Values are shown as mean ± standard deviation, median (interquartile range) or number (percentage).

P values refer to comparisons between the derivation group and the validation group.

### Comparison of parenchymal volume estimates obtained by contrast-enhanced CT and unenhanced CT

**Figure 1:**
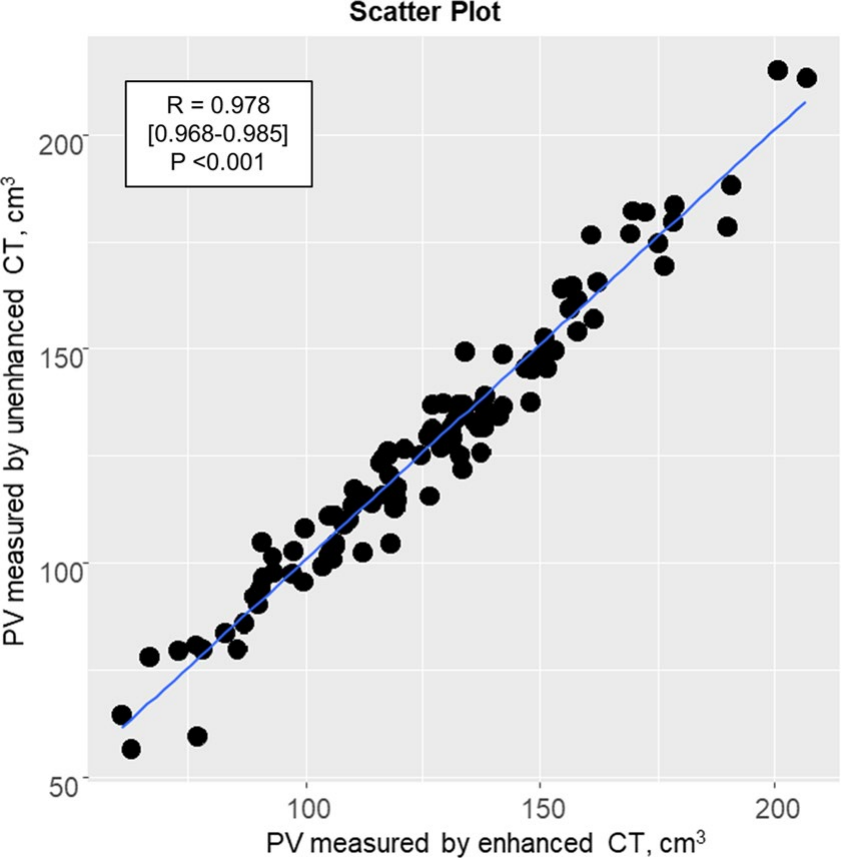
Correlation between parenchymal volume estimates obtained using enhanced and unenhanced CT imaging. The correlation shows excellent agreement between parenchymal volume estimates obtained using enhanced and unenhanced CT imaging. Abbreviation: CT, computed tomography; PV, parenchymal volume.

The value of the cortical volume based on enhanced CT was 126.3 ± 30.1 cm^3^ per kidney and that based on unenhanced CT was 127.2 ± 31.0 cm^3^ per kidney. As shown in Fig. 1, there was a strong correlation between parenchymal volume values obtained by contrast-enhanced CT and unenhanced CT (R = 0.978 [95% confidence interval 0.968–0.985], P < 0.001). Since unenhanced CT avoids the concerns of contrast in kidney disease patients, further analysis on parenchymal volume was limited to unenhanced CT.

### Univariate and multivariate analyses of factors associated with cortical volume

The univariate and multivariate analyses of factors associated with cortical volume estimates obtained using enhanced CT images are shown in Table 2. The mean value of the cortical volume was 89.3 ± 23.4 cm^3^ per kidney. In univariate analyses, neither diabetes nor hypertension were significantly associated with cortical volume, and thus were excluded from the models. Based on multivariate analyses, we created four models by using sequential regression to estimate cortical volume as follows (note that in the equations CV refers to cortical volume and PV refers to parenchymal volume): (i) Equation 1, estimated CV (cm^3^) = −17.1 (intercept) + 0.15 × eGFR (mL/min/1.73 m^2^) + 0.076 × body height (cm) + 0.67 × PV (cm^3^); (ii) Equation 2, estimated CV (cm^3^) = 35.4 (intercept) − 0.2 × age (year) + 4.77 (if male) − 13.2 × log [Cr (mg/dL)] − 0.14 × body height (cm) + 0.67 × PV (cm^3^); (iii) Equation 3, estimated CV (cm^3^) = −15.5 (intercept) + 0.17 × eGFR (mL/min/1.73 m^2^) + 0.25 × body weight (kg) + 0.62 × PV (cm^3^); (iv) Equation 4, estimated CV (cm^3^) = 0.4 (intercept) − 0.12 × age (year) + 1.29 (if male) − 15.8 × log[Cr (mg/dL)] + 0.23 × body weight (kg) + 0.63 × PV (cm^3^). By adding all these variables and using the stepwise selection method with Akaike information criteria, the same equation as Equation 3 was created. We also created a regression equation of ‘model 0’ using only PV as an explanatory variable: Equation 0, estimated CV (cm^3^) = −1.3 (intercept) + 0.71 × PV (cm^3^). In each regression equation, the estimated cortical volume showed strong correlations with measured cortical volume with small root mean squared error (RMSE) and mean absolute error (MAE), regardless of whether they were donors or diseased patients as shown in Table 3

**Table 2:** Correlation and multiple linear regression for renal cortical volume in the derivation group (N = 80).

	Univariate		Multivariate model 0			Multivariate model 1			Multivariate model 2			Multivariate model 3			Multivariate model 4		
	β coefficient (95%CI)	P value	β coefficient (95%CI)	P value	VIF	β coefficient (95%CI)	P value	VIF	β coefficient (95%CI)	P value	VIF	β coefficient (95%CI)	P value	VIF	β coefficient (95%CI)	P value	VIF
Age, year	−0.417 (−0.827, −0.008)	0.046	—	—	—	—	—	—	−0.209 (−0.378, −0.039)	0.016	1.14	—	—	—	−0.118 (−0.291, 0.055)	0.180	1.25
Gender, male	12.5 (2.0, 22.9)	0.020	—	—	—	—	—	—	4.78 (−2.49, 12.05)	0.195	3.16	—	—	—	1.29 (−4.68, 7.26)	0.67	2.24
log Cr, 10 log(mg/dL)	−49.7 (−79.5, −20.0)	0.0013	—	—	—	—	—	—	−13.2 (−30.6, 4.2)	0.135	2.10	—	—	—	−15.8 (−33.0, 1.4)	0.071	2.15
eGFR, mL/min/1.73 m^2^	0.656 (0.406, 0.905)	<0.001	—	—	—	0.152 (0.026, 0.278)	0.019	1.29	—	—	—	0.168 (0.048, 0.288)	0.007	1.28	—	—	—
Body height, cm	1.06 (0.41, 1.71)	0.0016	—	—	—	0.076 (−0.210, 0.363)	0.595	1.18	−0.136 (−0.543, 0.270)	0.51	2.43	—	—	—	—	—	—
Body weight, kg	0.98 (0.61, 1.35)	<0.001	—	—	—	—	—	—	—	—	—	0.250 (0.073, 0.428)	0.006	1.29	0.232 (0.009, 0.455)	0.042	1.98
Diabetes mellitus	1.8 (−12.7, 16.4)	0.803	—	—	—	—	—	—	—	—	—	—	—	—	—	—	—
Hypertension	4.3 (−6.4, 15.0)	0.427	—	—	—	—	—	—	—	—	—	—	—	—	—	—	—
PV plain, cm^3^	0.714 (0.645, 0.782)	<0.001	0.714 (0.645, 0.782)	<0.001	—	0.665 (0.584, 0.747)	<0.001	1.49	0.671 (0.585, 0.756)	<0.001	1.67	0.621 (0.540, 0.702)	<0.001	1.61	0.630 (0.541, 0.719)	<0.001	1.9
				Adjusted R^2^ = 0.845, AIC = 361			Adjusted R^2^ = 0.852, AIC = 361			Adjusted R^2^ = 0.856, AIC = 359			Adjusted R^2^ = 0.866, AIC = 352			Adjusted R^2^ = 0.875, AIC = 354	

Footnote: Common logarithm was used for logarithmic transformation. Abbreviations: AIC, Akaike information criteria; CI, confidence interval; Cr, creatinine; eGFR, estimated glomerular filtration rate; PV, parenchymal volume, VIF, variance inflation factor.

**Table 3:** Correlation, RMSE and MAE in the derivation group (N = 80).

Equation	All subjects (N = 80)					Donor subjects (N = 35)					Diseased subjects (N = 45)				
	R coefficient	95% CI	P value	RMSE, cm^3^	MAE, cm^3^	R coefficient	95% CI	P value	RMSE, cm^3^	MAE, cm^3^	R coefficient	95% CI	P value	RMSE, cm^3^	MAE, cm^3^
Eq. 0	0.920	0.878–0.948	<0.001	9.34	7.44	0.889	0.790–0.943	<0.001	9.88	7.80	0.941	0.895–0.968	<0.001	8.89	7.16
Eq. 1	0.926	0.887–0.952	<0.001	9.01	7.18	0.892	0.795–0.945	<0.001	9.52	7.65	0.943	0.899–0.969	<0.001	8.59	6.81
Eq. 2	0.930	0.892–0.954	<0.001	8.80	7.15	0.902	0.814–0.950	<0.001	9.02	7.44	0.942	0.897–0.968	<0.001	8.62	6.93
Eq. 3	0.933	0.897–0.957	<0.001	8.58	6.88	0.903	0.814–0.950	<0.001	9.09	7.27	0.949	0.909–0.972	<0.001	8.17	6.57
Eq. 4	0.933	0.898–0.957	<0.001	8.58	6.99	0.903	0.815–0.950	<0.001	9.00	7.23	0.947	0.906–0.97	<0.001	8.24	6.77

Footnote: (i) Eq. 0, estimated CV (cm^3^) = −1.3 (intercept) + 0.71 × PV (cm^3^);

(ii) Eq. 1, estimated CV (cm^3^) = −17.1 (intercept) + 0.15 × eGFR (mL/min/1.73 m^2^) + 0.076 × body height (cm) + 0.67 × PV (cm^3^);

(iii) Eq. 2, estimated CV (cm^3^) = 35.4 (intercept) − 0.2 × age (year) + 4.77 (if male) − 13.2 × log[Cr (mg/dL)] − 0.14 × body height (cm) + 0.67 × PV (cm^3^);

(iv) Eq. 3, estimated CV (cm^3^) = −15.5 (intercept) + 0.17 × eGFR (mL/min/1.73 m^2^) + 0.25 × body weight (kg) + 0.62 × PV (cm^3^);

(v) Eq. 4: estimated CV (cm^3^) = 0.4 (intercept) − 0.12 × age (year) + 1.29 (if male) − 15.8 × log[Cr (mg/dL)] + 0.23 × body weight (kg) + 0.63 × PV (cm^3^).

Abbreviations: CI, confidence interval; MAE, mean absolute error; RMSE, root mean squared error; CV cortical volume; PV, parenchymal volume.

.

We performed additional analyses to confirm that the severity of renal risk factors, including diabetes and hypertension, were not associated with CV. Two-way ANOVA between CKD category in relation to diabetes or hypertension was performed to examine whether there was any influence on CV depending on the stage of CKD (Supplemental Tables S3 and S4). The CV decreased in accordance with CKD category, but there were no significant effects or interactions between CKD category in relation to diabetes or hypertension. Further, no significant correlations were found between duration of diabetes or hypertension and CV (Supplemental Table S5).

### Analyses in the validation group

**Figure 2:**
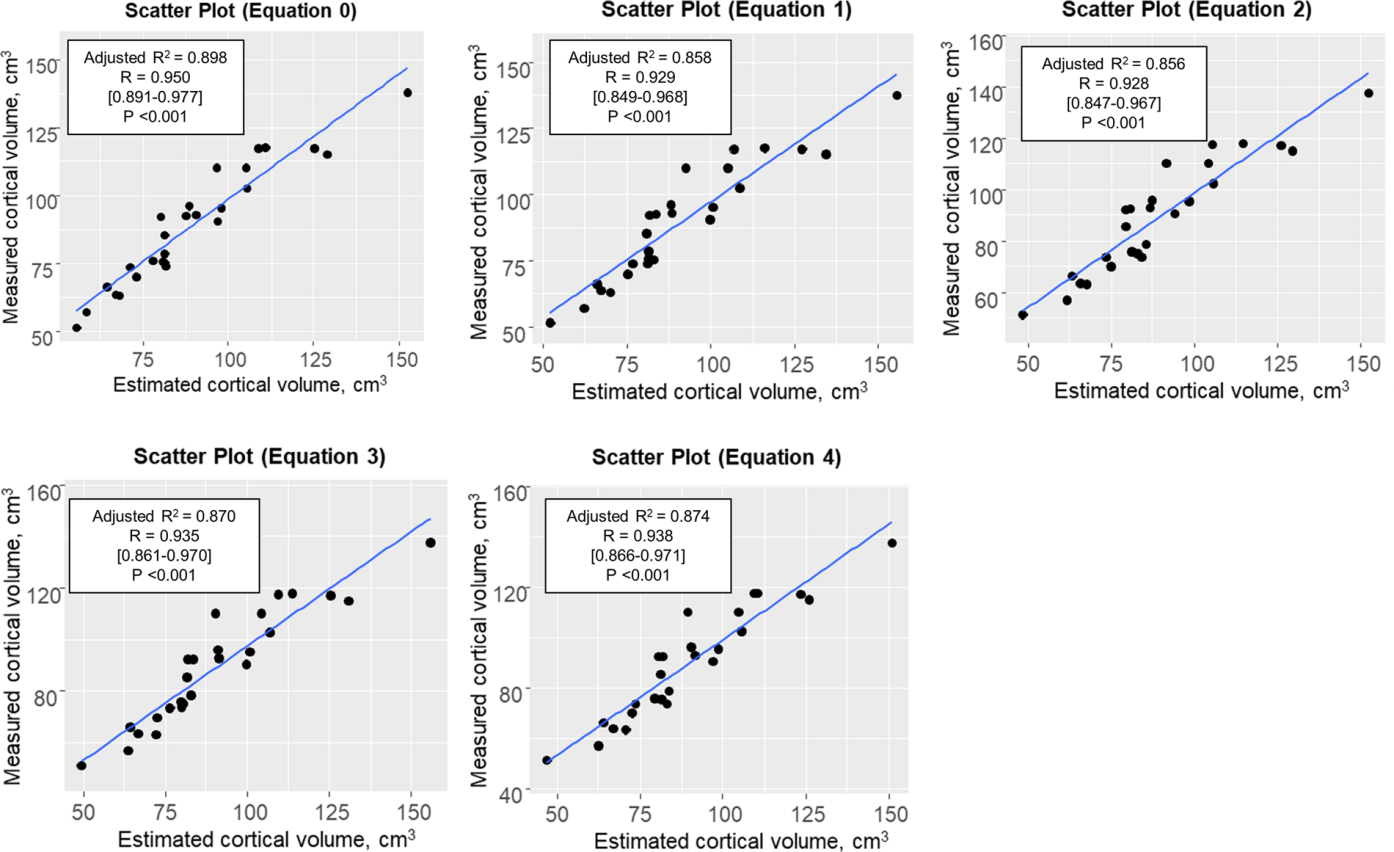
Correlation for cortical volume in the validation group. Correlation analyses showed high degrees of consistency between the cortical volume estimates obtained using unenhanced CT and contrast-enhanced CT in the validation group.

The validities of the estimated values of cortical volume obtained using the five equations were confirmed using the validation group. The estimated cortical volumes based on each estimation equation model showed strong correlations with the measured cortical volumes obtained using contrast-enhanced CT (Fig. 2). The RMSE and MAE of the estimated cortical volume based on Equation 0 were the lowest, but both of these error values were sufficiently low for all five regression equation models (Table 4).

**Table 4:** RMSE, MAE, absolute bias and relative bias of estimated cortical volume in the validation group (N = 27).

Equation	RMSE, cm^3^	MAE, cm^3^	Absolute bias, cm^3^	Relative bias, %
Eq. 0	7.22	6.09	−0.28	−0.77
Eq. 1	8.61	7.17	−0.76	−1.23
Eq. 2	8.33	7.05	−1.83	−2.44
Eq. 3	8.14	6.65	−0.37	−0.78
Eq. 4	7.65	6.37	−1.31	−1.78

Footnote: (i) Eq. 0, estimated CV (cm^3^) = −1.3 (intercept) + 0.71 × PV (cm^3^);

(ii) Eq. 1, estimated CV (cm^3^) = −17.1 (intercept) + 0.15 × eGFR (mL/min/1.73 m^2^) + 0.076 × body height (cm) + 0.67 × PV(cm^3^);

(iii) Eq. 2, estimated CV (cm^3^) = 35.4 (intercept) − 0.2 × age (year) + 4.77 (if male) − 13.2 × log[Cr (mg/dL)] − 0.14 × body height (cm) + 0.67 × PV(cm^3^);

(iv) Eq. 3, estimated CV (cm^3^) = −15.5 (intercept) + 0.17 × eGFR (mL/min/1.73 m^2^) + 0.25 × body weight (kg) + 0.62 × PV (cm^3^);

(v) Eq. 4: estimated CV (cm^3^) = 0.4 (intercept) − 0.12 × age (year) + 1.29 (if male) − 15.8 × log[Cr (mg/dL)] + 0.23 × body weight (kg) + 0.63 × PV (cm^3^).

Abbreviations: CI, confidence interval; MAE, mean absolute error; RMSE, root mean squared error.

Stratified analysis was performed to determine whether equation 0 based on PV alone can be applied to subgroups categorized based on various clinical conditions (Supplemental Table S6). None of the clinical conditions examined including age, gender, CKD, diabetes or hypertension modified the close correlations between measured CV and estimated CV based on equation 0.

The applicability of the present method was confirmed among the additionally recruited patients with advanced CKD who were excluded because of deficit of venous phase CT images (N = 16). In each regression equation, the estimated CV showed significant correlations with measured CV, with small RMSE and MAE (Supplemental Tables S7 and S8).

### Comparisons of nephron number estimates obtained using enhanced and unenhanced CT images

In the donor group, nephron number was estimated using cortical volume values obtained using the equation models shown above and glomerular density estimates obtained using biopsy-based stereology (N = 44). Five cases were excluded due to insufficient numbers of glomeruli in the biopsies. The non-sclerotic glomerular density and the total glomerular density were 13.5 ± 4.7/mm^3^ and 15.0 ± 5.9/mm^3^, respectively. The estimated nephron number obtained with each model was compared to that obtained using enhanced CT-measured cortical volume. As shown in Table 5 and Supplemental Fig. S2, there were strong correlations between the two sets of nephron number estimates, with minimal RMSE and MAE. There were also strong agreements between the two methods for estimates of total nephron number and globally sclerotic glomeruli.

**Table 5:** Nephron number in the donor group for whom a renal biopsy sample was available (N = 44).

Equation	Nephron number based on estimated CV, mean ± SD × 10^3^/kidney	Nephron number based on measured CV, mean ± SD × 10^3^/kidney	R value	95% CI	P value	RMSE, × 10^3^/kidney	MAE, × 10^3^/kidney
Equation	Total nephron number including sclerotic glomeruli based on estimated CV, mean (SD) × 10^3^/kidney	Total nephron number including sclerotic glomeruli based on measured CV, mean (SD) × 10^3^/kidney	R value	95% CI	P value	RMSE, × 10^3^/kidney	MAE, × 10^3^/kidney
Eq. 0	636 ± 210	648 ± 224	0.955	0.918–0.975	<0.001	67	49
Eq. 1	655 ± 219	648 ± 224	0.951	0.912–0.973	<0.001	69	53
Eq. 2	646 ± 215	648 ± 224	0.958	0.924–0.977	<0.001	64	50
Eq. 3	652 ± 216	648 ± 224	0.958	0.924–0.977	<0.001	64	50
Eq. 4	647 ± 215	648 ± 224	0.961	0.929–0.979	<0.001	61	48
Eq. 0	700 ± 240	712 ± 245	0.957	0.922–0.976	<0.001	72	53
Eq. 1	721 ± 248	712 ± 245	0.954	0.916–0.975	<0.001	75	58
Eq. 2	711 ± 242	712 ± 245	0.959	0.926–0.978	<0.001	69	55
Eq. 3	718 ± 244	712 ± 245	0.959	0.926–0.978	<0.001	69	55
Eq. 4	712 ± 242	712 ± 245	0.962	0.931–0.979	<0.001	66	53

Footnote: (i) Eq. 0, estimated CV (cm^3^) = −1.3 (intercept) + 0.71 × PV (cm^3^); (ii) Eq. 1, estimated CV (cm^3^) = −17.1 (intercept) + 0.15 × eGFR (mL/min/1.73 m^2^) + 0.076 × body height (cm) + 0.67 × PV (cm^3^);

(iii) Eq. 2, estimated CV (cm^3^) = 35.4 (intercept) − 0.2 × age (year) + 4.77 (if male) − 13.2 × log[Cr (mg/dL)] − 0.14 × body height (cm) + 0.67 × PV (cm^3^);

(iv) Eq. 3, estimated CV (cm^3^) = −15.5 (intercept) + 0.17 × eGFR (mL/min/1.73 m^2^) + 0.25 × body weight (kg) + 0.62 × PV (cm^3^);

(v) Eq. 4: estimated CV (cm^3^) = 0.4 (intercept) − 0.12 × age (year) + 1.29 (if male) − 15.8 × log[Cr (mg/dL)] + 0.23 × body weight (kg) + 0.63 × PV (cm^3^).

Abbreviations: CI, confidence interval; CV, cortical volume; MAE, mean absolute error; RMSE, root mean squared error; TNN, total nephron number.

## Discussion

The aim of this study was to produce a model to estimate renal cortical volume using parenchymal volume measured using unenhanced CT imaging. Body size (body weight or body height), renal function (eGFR or Cr), diabetes, hypertension, age, gender, and parenchymal volume were selected as candidate variables for the regression equation models. In univariate analyses, neither diabetes nor hypertension were significantly associated with cortical volume and were therefore excluded from explanatory variables. Using multivariate analyses, we produced four regression equation models for the estimation of cortical volume using unenhanced CT images. We also created a simple model with only parenchymal volume as an explanatory variable for comparison. The cortical volume values obtained by the models showed strong correlations with the measured cortical volumes obtained using the enhanced CT-based method, for both kidney donors and patients with disease. In addition, we confirmed the validity of the models in a validation group. These results suggest that the regression equations produced in this study can produce accurate and precise estimates of cortical volume.

In this study, the mean value for measured cortical volume was 89.3 cm^3^ per kidney. This value is consistent with, although somewhat lower than previous reports of cortical volume of approximately 90 to 120 cm^3^ per kidney^3,4,7^. Previous studies have shown that a range of factors influence cortical volume including age, gender, renal function, diabetes, hypertension, body size, and renal morphological parameters^3,4,6,7,13,14^. In this study, we tried to produce a model by applying these factors as candidate explanatory variables for cortical volume. First, we selected Cr as a renal function parameter. We additionally created a model based on eGFR without age, gender or Cr because eGFR is a renal function parameter calculated from these parameters, and age-related decreases in cortical volume have been reported to correlate with GFR^4,5^. In fact, in the present study, no single component of eGFR (age, gender, serum Cr level) was more strongly correlated with the enhanced CT-measured cortical volume than eGFR itself in univariate regression analyses. We therefore concluded that eGFR is applicable to one of the models for estimating cortical volume. Second, both body height and body weight are reported as factors related to cortical volume^3^. The present findings confirmed these relationships. Third, renal morphological metrics such as renal thickness, width and length have been examined as explanatory factors for cortical volume in previous studies^1,2,15^. In this study, parenchymal volume was used as an explanatory variable, since we considered it may be morphologically more homologous to cortical volume than thickness and length measurements. Our results clearly demonstrated that parenchymal volume was the strongest variable in the determination of cortical volume, though other variables such as body size and renal function metrics were also identified as significant determinants of cortical volume.

We hypothesized that clinical characteristics could be combined with parenchymal volume to better estimate cortical volume. We therefore produced five variations of the regression model based on age, gender, renal function, body size and parenchymal volume. Although the equation based on age, gender, log [Cr], body weight and parenchymal volume was statistically the most suitable model of the five equations, the validity of each model was confirmed. In the validation group, the equation that used only parenchymal volume had the highest fitness and the lowest value of Akaike information criteria. However, the differences in RMSE and MAE were up to 1.65 and 1.32 cm^3^ (within 2% to the cortex) among the models, suggesting that this difference can be clinically ignored. These results suggest that clinical characteristics combined with parenchymal volume did not meaningfully improve the equation over parenchymal volume alone. This is probably due to the fact that parenchymal volume had the strongest influence in each model.

In clinical settings, body weight can change depending on the etiology of the renal disease, especially in such cases as nephrotic syndrome with systemic edema. Such variability could influence an estimate of cortical volume if body weight was included in an equation model. Thus, the body size parameters in these equation models should be modified depending on the type of renal disease, and possibly a model using body height or a model that uses only parenchymal volume would be more appropriate in some cases.

Although the number of nephrons does not increase in humans after term birth, each nephron has the potential to undergo hypertrophy and thus contribute more to renal function. In subjects with low nephron number, enlargement of the renal cortex due to compensatory hypertrophy of individual nephrons occurs in association with increasing demands such as normal body growth and catch-up growth^16,17^. Similar compensatory changes may occur in response to nephron loss in patients with chronic renal diseases^18^. Thus, there remains an issue of difficulty to interpret the number of nephrons in an individual based solely on knowledge of cortical volume. In fact, previous reports of cortical volume did not consistently find correlations with either nephron number or renal outcomes^19,20^.

Differences in nephron number may also be indicative of differences in renal functional reserve between individuals with similar clinical characteristics at the time of diagnosis of kidney disease, which is recognized as one cause of divergent renal outcomes^21,22^. Although differences in nephron number have been extensively investigated in different racial and socioeconomic groups, until recently, data for Asian populations including Japanese subjects was lacking. Recently, we estimated nephron number in Japanese subjects using the gold-standard disector/fractionator method and reported that values were significantly lower than in other races^12^. Estimating the number of individual nephrons is considered to be clinically useful for assessing the future progression of kidney diseases. However, the disector/fractionator method, which is the standard method for accurately measuring the total number of nephrons, requires a great deal of labor and time and cannot be applied to living subjects^23^. In the present study, we confirmed that the difference between enhanced or unenhanced CT-based methods for estimating nephron number was minimal. Interestingly, greater variation was found in estimates of subject glomerular density than cortical volume. Thus, our results suggest that the unenhanced CT method can be used to estimate cortical volume as well as nephron number. This approach may therefore allow us to estimate nephron number in renal disease patients who are often not suitable for contrast-enhanced CT imaging.

Limitations of this study include the relatively small number of patients with renal impairment since nearly half of the subjects were living kidney donors. Patients with heavy proteinuria tend to be avoided for using contrast agents and may not be included in our subjects. While PV can be used to estimate CV, PV is an imperfect surrogate for CV as some clinical factors associate with CV differently than PV^4^. This is the “trade-off” with some modest loss of accuracy but avoiding contrast nephropathy. Further, our equation may not be applicable in advanced CKD, where much more severely disproportionate loss of CV (cortical thinning) occurs. In addition, the results of this study need further study in children and in other races than Japanese adult subjects. However, we expect that with minor modifications this methodology would be applicable to other subjects.

In conclusion, this study provides regression equation models to estimate renal cortical volume by using unenhanced CT images. Estimation of cortical volume by this method may be useful for the estimation of individual nephron number as well as prediction of renal outcomes, with a possible application for patients with renal disease.

## Methods

### Patient selection

In this study, we reviewed images and clinical data from diseased patients and living kidney donors in our department from 2007 to 2017 who underwent abdominal contrast-enhanced CT, in which whole kidney images were included. All patients were simultaneously subjected to unenhanced and contrast-enhanced CT examinations at the same time. Kidney donors were selected according to the Amsterdam Forum guidelines^24^. 107 subjects including 49 donors and 58 patients were recruited, and they were randomly assigned into the derivation group (N = 80) for model creation and the validation group (N = 27). This study was approved by the ethics review board of the Jikei University School of Medicine [30–229 (9250)]. Since this study used only existing clinical data, we provided participants with information about the opportunity to opt out and obtained a waiver of informed consent.

### Morphological measurements

**Figure 3:**
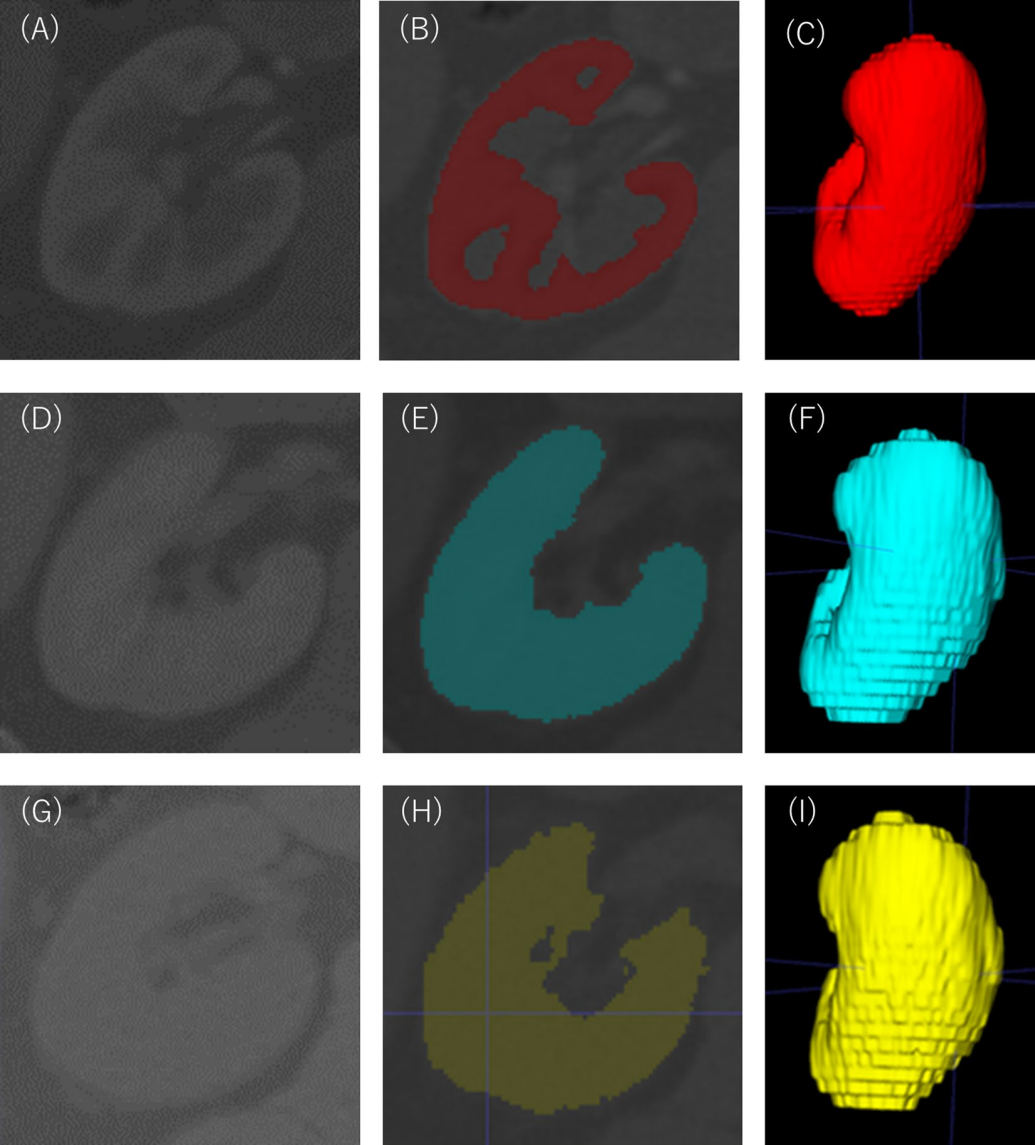
Measurement of renal cortical volume and renal parenchymal volume. A three-dimensional cortical image was semi-automatically constructed from the contrast-enhanced images obtained in the arterial phase after contrast media infusion (**A**–**C**). A three-dimensional parenchymal image was semi-automatically constructed from the contrast-enhanced images obtained in the venous phase after contrast media infusion (**D**–**F**). As with the venous phase, a three-dimensional image was semi-automatically constructed from the image of specific density areas of kidney obtained from unenhanced CT images (**G**–**I**). Renal artery, renal vein, renal pelvis, ureter, renal sinus, fat in renal sinus, adjacent tissue and adjacent organs were excluded from all the created images.

CT images of kidneys were acquired by the Aquilion Prime (Toshiba Medical Systems, Otawara, Japan), the Definition AS+ (Siemens, Munich, Germany) or the Definition flash (Siemens, Munich, Germany). The thickness of the obtained image was in the range of 3.0 to 5.0 mm. The arterial phase and venous phase images were obtained 30 and 90 seconds after infusion of intravenous contrast, respectively. Renal cortical volumes were measured as previously described^9,11^ using software (ITK-SNAP version 1.1, University of Pennsylvania, Philadelphia, PA, www.itksnap.org) to semi-automatically segment the cortex and medulla from transverse images obtained during the arterial phase of the CT angiogram (Fig. 3A–C). Parenchymal volume was measured using images obtained during the venous phase (Fig. 3D–F) and the unenhanced phase of the CT angiogram (Fig. 3G–I).

**Figure 4:**
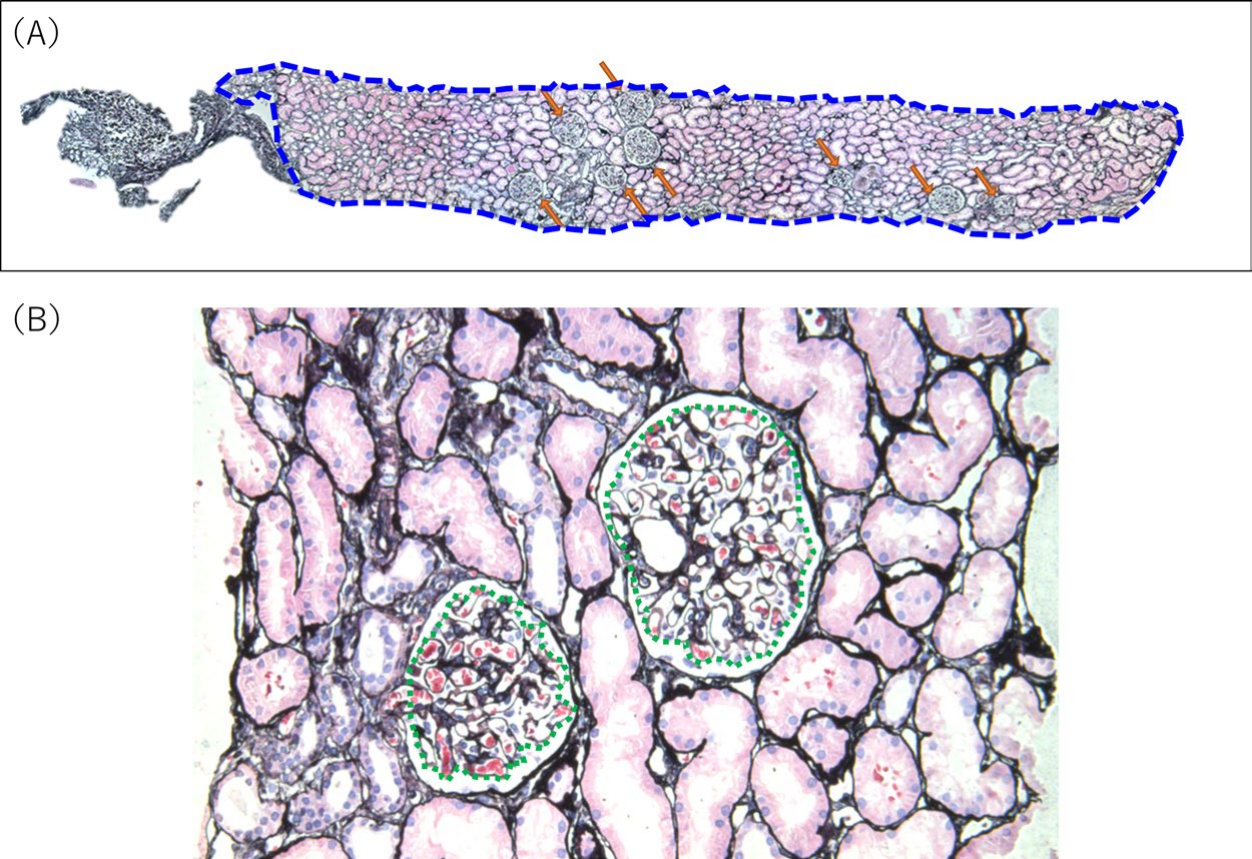
Renal biopsy morphometry. (**A**) The cortical area was measured by outlining the circumference of the cortical sample in the biopsy (blue dashed line). The number of non-sclerotic glomeruli in the cortex was counted (orange arrows). Periodic acid–methenamine silver staining, original magnification × 50. (**B**) Glomerular tuft areas were outlined (green dotted line). The mean area of all non-sclerotic glomerular tufts in the biopsy was used to calculate mean glomerular volume. Periodic acid–methenamine silver staining, original magnification × 400.

In the donor group, nephron number was estimated by enhanced or unenhanced CT-based methods combined with glomerular numerical density obtained through stereological analysis of an implantation biopsy as reported by Denic *et al*.^9^. Donors with less than five glomeruli in their biopsy were excluded from the analysis of nephron number. Nephron number was defined as the number of non-sclerotic glomeruli (NSG) and was calculated by multiplying total cortical volume (mm^3^) by the numerical density of NSG (number per mm^3^ of cortex) (Fig. 4A). These values were divided by 2 (per kidney), by 1.43 (to correct for tissue volume shrinkage due to paraffin embedding), and by 1.268 (to correct for volume shrinkage due to loss of tissue perfusion pressure)^9,11^.

In biopsies, the combined areas of all glomerular capillary tufts and the area of renal cortex were measured using a computerized image analyzer (Leica IM500, Leica Microsystems, Germany). Glomerular tuft area was defined as the area of the outer capillary loops of the tuft (Fig. 4B). Glomeruli with global glomerulosclerosis were excluded from the analysis.

The total number of NSG was obtained by summing the numbers of complete and partial NSG (counted as 0.5 NSG). The mean area of NSG per biopsy was obtained by dividing the total area of all NSG by the total number of NSG. NSG density (per mm^3^ of cortex) was calculated using the Weibel and Gomez stereological method as follows^25^: ${\rm{NSG}}\,{\rm{density}}=\frac{1}{\beta }\times \sqrt[2]{\frac{{(\frac{TotalnumberofNSG}{Areaofcortex})}^{3}}{\frac{Total\,area\,of\,NSG}{Area\,of\,cortex}}}$, where β is a dimensionless shape coefficient (β = 1.382 for spheres) (Fig. 3). The sclerotic glomerular density (per mm^3^ of cortex) was also calculated: ${\rm{sclerotic}}\,{\rm{glomerular}}\,{\rm{density}}\,=$ $\frac{1}{\beta }\times \,\sqrt[2]{\frac{{(\frac{Totalnumberofscleroticglomeruli}{Areaofcortex})}^{3}}{\frac{Total\,area\,of\,sclerotic\,glomeruli}{Area\,of\,cortex}}}$. Total glomerular density (per mm^3^ of cortex) was the sum of NSG density and sclerotic glomerular density. We previously reported nephron number estimated by enhanced CT-based methods in the same donor group^11^.

### Measurements of risk factors

Blood pressure was measured with the subject resting in a sitting position. Serum creatinine (Cr) was measured using the enzymatic method. These measured values, together with other blood laboratory values, body height and body weight were the latest available data before CT imaging. Hypertension was defined as a systolic blood pressure of >140 mmHg and/or diastolic blood pressure of >90 mmHg, or the use of antihypertensive medications. The estimated glomerular filtration rate (eGFR) was calculated from serum Cr using a modified equation for the GFR of Japanese individuals: eGFR = 194 × age^-0.287^ × Cr^-1.094^ (×0.739 if female)^26^. Chronic kidney disease (CKD) stages were defined as follows: G1, GFR ≥ 90 mL/min/1.73 m^2^; G2, GFR 60–89 mL/min/1.73 m^2^; G3, eGFR 30–59 mL/min/1.73 m^2^; G4, 15–29 mL/min/1.73 m^2^; G5, GFR < 15 mL/min/1.73 m^2^. Elderly was defined as 60 years and older.

### Statistical analysis

Continuous variables were assessed for normality both visually (normal probability plot and histogram) and by inferential statistics (Shapiro–Wilk tests) and are presented as mean ± standard deviation. Serum Cr was shown as median (interquartile range) in the clinical characteristics and was naturally log-transformed in the regression analysis due to the skewed distributions. Categorical variables were summarized by the frequency. For comparisons between the derivation group and the validation group, an unpaired t-test for continuous variables, a Mann-Whitney test for serum Cr, and a χ^2^ test for categorical variables were used.

Correlations were assessed using Pearson’s correlation coefficient. Previously reported or clinically relevant parameters were subjected to univariate or multivariate linear regression analyses in order to determine the model of estimated cortical volume. In addition to age, gender and renal function, height and weight were adopted as body size factors as an explanatory variable reported to be related to cortical volume. The absolute bias, which is the mean differences between the measured cortical volume and the estimated cortical volume, was calculated. The relative bias, which is the division of the mean differences between the measured cortical volume and the estimated cortical volume, was calculated. Two-sided P values of <0.05 were considered statistically significant. All statistical analyses were performed using EZR (Saitama Medical Center, Jichi University, Saitama, Japan), which is based on the R software package (The R Foundation for Statistical Computing, version 3.2.2)^27^. Other R packages were used for analyses as follows: (i) ‘Metrics’ package for calculating the RMSE and the MAE, (ii) ‘ggplot2’ package for drawing scatter plots and regression lines.

### Ethical approval and informed consent

This study was approved by the ethics review board of the Jikei University School of Medicine [30-229 (9250)]. Since this study used only existing clinical data, we obtained a waiver of informed consent in accordance with relevant guidelines and regulations.

## Supplementary information

Supplementary information

## Data Availability

These data in the present study are available from the corresponding author on reasonable request.
