# Supplementary material for: Estimation of nephron number in living humans by combining unenhanced computed tomography with biopsy-based stereology
Source: Sci Rep. 2019 Oct 7;9:14400. doi: 10.1038/s41598-019-50529-x (PMC6779756; doi:10.1038/s41598-019-50529-x)
Supplement: Supplementary file 1 — Supplementary information [file 41598_2019_50529_MOESM1_ESM.docx]

**Supplemental information**

**Estimation of nephron number in living humans by combining unenhanced computed tomography with biopsy-based stereology**

Takaya Sasaki^1^, Nobuo Tsuboi^1*^, Yusuke Okabayashi^1^, Kotaro Haruhara^1^, Go Kanzaki^1^, Kentaro Koike^1^, Akimitsu Kobayashi^1^, Izumi Yamamoto^1^, Sho Takahashi^2^, Toshiharu Ninomiya^3^, Akira Shimizu^4^, Andrew D. Rule^5^, John F. Bertram^6^, and Takashi Yokoo^1^.

^1^Division of Nephrology and Hypertension, Department of Internal Medicine, The Jikei University School of Medicine, Tokyo, Japan, ^2^ Clinical Research Support Center, The Jikei University School of Medicine Tokyo, Japan, ^3^ Department of Epidemiology and Public Health, Graduate School of Medical Sciences, Kyushu University, Fukuoka, Japan, ^4^ Department of Analytic Human Pathology, Nippon Medical School, Tokyo, Japan, ^5^Division of Nephrology and Hypertension, Mayo Clinic, Rochester, Minnesota, ^6^ Department of Anatomy and Developmental Biology and Biomedical Discovery Institute, Monash University, Melbourne, Australia.

**Supplemental Figure S1.** **Correlations for cortical volume in the derivation group**

**
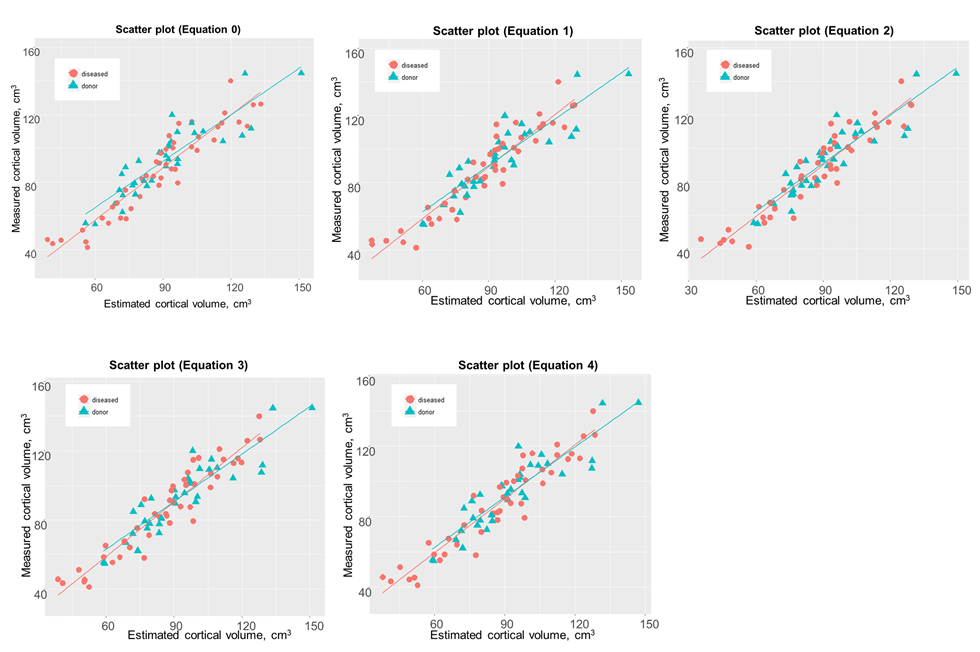
**

There were strong correlations between the estimated values of cortical volume obtained using contrast-unenhanced CT and the measured cortical volumes obtained using enhanced CT, in both the donor and diseased groups of the derivation group.

**Supplemental Figure S2. Correlation analyses for total nephron number in the donor subjects of the derivation group**

**
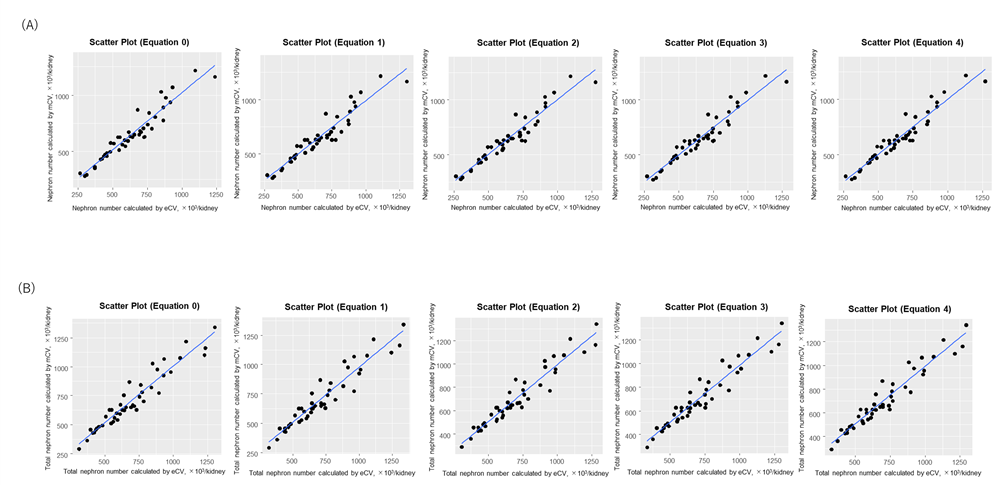
**

Strong correlations were found between nephron number estimates based on estimated cortical volume obtained using unenhanced CT and measured cortical volume obtained using enhanced CT in the donor group (**A**). Similar outcomes were found for estimates of total nephron number, including globally sclerotic glomeruli (**B**).

Abbreviations: eCV, estimated cortical volume; mCV, measured cortical volume.

| **Supplemental Table S1. Indications for performing contrast-enhanced CT in the diseased group** | |
| --- | --- |
| Indications | Number of patients |
| Secondary hypertension | 17 |
| Aortic aneurysm | 10 |
| Malignant diseases | 8 |
| Systemic inflammatory diseases | 6 |
| Acute abdomen | 4 |
| Donor candidate | 3 |
| Recipient candidate | 2 |
| Deep vein thrombosis | 2 |
| Fever of unknown origin | 2 |
| Liver abscess | 1 |
| Retroperitoneal abscess | 1 |
| Not available | 2 |

| **Supplemental Table S2. Clinical characteristics in the derivation and validation groups.** | | | |
| --- | --- | --- | --- |
|  | Derivation group | Validation group |  |
|  | N = 80 | N = 27 | P value |
| Donor, N (%) | 35 (43.8) | 14 (51.9) | 0.5 |
| Age, years | 58.1 ± 12.9 | 62.0 ±11.4 | 0.2 |
| Male, N (%) | 36 (45.0) | 14 (51.9) | 0.7 |
| Hypertension, N (%) | 37 (46.2) | 17 (63.0) | 0.2 |
| Duration of hypertension, years | 9 (4-12) | 9.5 (3.5-8.75) | >0.9 |
| Diabetes mellitus, N (%) | 13 (16.2) | 4 (14.8) | >0.9 |
| Duration of diabetes mellitus, years | 3 (2-6) | 7.5 (5.75-8.75) | 0.3 |
| Obesity, N (%) | 29 (36.2) | 8 (29.6) | 0.6 |
| CKD stage G1 and G2, N (%) | 48 (60.0) | 17 (63) | >0.9 |
| CKD stage G3, N (%) | 27 (33.8) | 9 (33.3) |  |
| CKD stage G4 and G5, N (%) | 5 (6.2) | 1 (3.7) |  |
| Body weight, kg | 61.4 ± 12.6 | 60.7 ±10.5 | 0.8 |
| Body height, cm | 161.6 ± 7.9 | 161.8 ± 8.1 | 0.9 |
| Cr, mg/dL | 0.80 (0.70-1.00) | 0.70 (0.63-1.08) | 0.3 |
| eGFR, ml/min/1.73m^2^ | 63.3 ± 18.7 | 67.8 ± 23.3 | 0.3 |
| Albumin, mg/dL | 3.95 ± 0.63 | 4.09 ± 0.37 | 0.3 |
| Serum total cholesterol, mg/dL | 201 ± 40 | 209 ± 38 | 0.4 |
| Hemoglobin A1c, % | 5.6 ± 0.9 | 5.6 ± 0.5 | 0.9 |
| Abbreviations: Cr, creatinine; eGFR, estimated glomerular filtration rate.  Values are shown as mean ± standard deviation, median (interquartile range) or number (percentage).  P values refer to comparisons between the Derivation Group and the Validation Group. | | | |

| **Supplemental Table S3**. Effect of CKD category and diabetes on CV in the derivation group (n = 80) | | |
| --- | --- | --- |
| Factor | F value | P value |
| CKD category | 10.14 | 0.002 |
| Diabetes mellitus | 0.03 | 0.87 |
| Interaction of CKD category and diabetes mellitus | 2.26 | 0.14 |
| Abbreviations: CKD, chronic kidney disease; CV, cortical volume. | | |

| **Supplemental Table S4**. Effect of CKD category and hypertension on CV in the derivation group (n = 80) | | |
| --- | --- | --- |
| Factor | F value | P value |
| CKD category | 11.75 | <0.001 |
| Hypertension | 1.24 | 0.27 |
| Interaction of CKD category and Hypertension | 0.35 | 0.70 |
| Abbreviations: CKD, chronic kidney disease; CV, cortical volume. | | |

| **Supplemental Table S5. Correlation between duration of diabetes or hypertension with CV in the derivation group (n = 80)** | | |
| --- | --- | --- |
|  | R coefficient | P value |
| Duration of hypertension (n = 37) | -0.03 | 0.87 |
| Duration of diabetes mellitus (n = 13) | -0.07 | 0.81 |
| Abbreviations: CV, cortical volume. | | |

| **Supplemental Table S6. Stratified analysis of the correlation between measured CV and estimated CV based on equation 0 in the validation group (n = 27).** | | |
| --- | --- | --- |
|  | R coefficient | P value |
| Elderly (n = 16) | 0.943 | <0.001 |
| Not elderly (n = 11) | 0.974 | <0.001 |
| Men (n = 14) | 0.934 | <0.001 |
| Women (n = 13) | 0.967 | <0.001 |
| CKD (n = 10) | 0.973 | <0.001 |
| Without CKD (n = 17) | 0.967 | <0.001 |
| Diabetes (n = 4) | 0.981 | 0.019 |
| Without diabetes (n = 23) | 0.936 | <0.001 |
| Hypertension (n = 17) | 0.953 | <0.001 |
| Without hypertension (n = 10) | 0.944 | <0.001 |
| Abbreviations: CKD, chronic kidney disease; CV, cortical volume. | | |

| **Supplemental Table S7. Clinical and morphological characteristics of separately identified patients with CKD G4 and G5 (n = 16)** | |
| --- | --- |
| Age, years | 49.0 ± 21.2 |
| Male, n (%) | 11 (69) |
| Body weight, kg | 57.1 ± 13.7 |
| Body height, cm | 165.3 ± 11.0 |
| Cr, mg/dL | 8.16 ± 3.67 |
| eGFR, ml/min/1.73m^2^ | 7.44 ± 3.50 |
| measured CV, cm^3^ | 46.1 ± 25.8 |
| measured PV (unenhanced), cm^3^ | 69.6 ± 35.7 |
| Abbreviations: Cr, creatinine; CV, cortical volume; eGFR, estimated glomerular filtration rate; PV, parenchymal volume. | |

| **Supplemental Table S8. Correlations and errors between measured CV and estimated CV in separately identified patients with CKD G4 and 5 (n = 16)** | | | | | | | |
| --- | --- | --- | --- | --- | --- | --- | --- |
| Equation | R value | 95% CI | P value | RMSE, cm^3^ | MAE, cm^3^ | Absolute bias, cm^3^ | Relative bias, % |
| Eq 0 | 0.96 | 0.885-0.986 | <0.001 | 7.42 | 6.15 | 2.32 | 7.48 |
| Eq 1 | 0.961 | 0.888-0.987 | <0.001 | 7.54 | 5.23 | -2.86 | -5.16 |
| Eq 2 | 0.957 | 0.877-0.985 | <0.001 | 8.96 | 6.12 | -5.16 | -11.23 |
| Eq 3 | 0.952 | 0.865-0.984 | <0.001 | 8.32 | 5.94 | -2.87 | -3.75 |
| Eq 4 | 0.956 | 0.876-0.985 | <0.001 | 10.44 | 7.65 | -7.39 | -16.82 |
| Footnote: (i) Eq 1, estimated CV (cm^3^) = -17.1 (intercept) + 0.15 × eGFR (mL/min/1.73m^2^) + 0.076 × body height (cm) + 0.67 × PV(cm^3^);  (ii) Eq 2, estimated CV (cm^3^) = 35.4 (intercept) - 0.2 × age (year) + 4.77 (if male) - 13.2 × log[Cr (mg/dL)] - 0.14 × body height (cm) + 0.67 × PV(cm3);  (iii) Eq 3, estimated CV (cm3) = -15.5 (intercept) + 0.17 × eGFR (mL/min/1.73m2) + 0.25 × body weight (kg) + 0.62 × PV (cm3);  (iv) Eq 4: estimated CV (cm3) = 0.4 (intercept) - 0.12 × age (year) + 1.29 (if male) - 15.8 × log[Cr (mg/dL)] + 0.23 × body weight (kg) + 0.63 × PV (cm3). Abbreviations: CI, confidence interval; CKD, chronic kidney disease; MAE, mean absolute error; RMSE, root mean squared error. | | | | | | | |
